# Integrated omics revealed the altered colonic microenvironment after inhibition of peripheral serotonin synthesis by LP533401

**DOI:** 10.1002/imo2.34

**Published:** 2024-10-14

**Authors:** Yidan Ling, Ziyu Liu, Shuibing Han, Haiqin Wu, Chunlong Mu, Weiyun Zhu

**Affiliations:** ^1^ Laboratory of Gastrointestinal Microbiology, College of Animal Science and Technology Nanjing Agricultural University Nanjing China; ^2^ National Center for International Research on Animal Gut Nutrition, National Experimental Teaching Demonstration Center of Animal Science Nanjing Agricultural University Nanjing China; ^3^ Department of Animal Science University of Arkansas Fayetteville Arkansas USA; ^4^ Department of Biochemistry and Molecular Biology, Cumming School of Medicine University of Calgary Calgary Canada

**Keywords:** colon, gut microbiome, metabolomics, metagenomics, serotonin, stem cells

## Abstract

Gut‐derived 5‐hydroxytryptamine (5‐HT), known as serotonin, plays a crucial role in regulating gastrointestinal functions. However, the impact of disruptions in gut‐derived 5‐HT synthesis on the early gut microbiome and intestinal microenvironment remains unclear. In this study, LP533401, an inhibitor targeting peripheral 5‐HT synthesis, was administered orally to neonatal rats starting at 4 days post‐birth. By day 11, inhibition of gut‐derived 5‐HT resulted in altered colonic morphology, characterized by increased crypt depth and reduced myenteric thickness. To investigate the mechanisms underlying these alterations, we employed a combination of metagenomics, mucosal transcriptome, and untargeted metabolomics on colonic samples. Metagenome profiling revealed an upregulation in the microbial two‐component system (ko02020) and tyrosine metabolism (ko00350), with minimal effects on taxa abundances. Transcriptome profiling analysis indicated the discriminant expression of genes enriched in pathogen infection‐responsive signaling (e.g., *Salmonella* and *Yersinia* infection) and the Wnt signaling pathway that affected stem cell proliferation. Consistent with increased crypt depth, marker genes related to cell proliferation were excessively activated. Metabolomics analysis indicated lower ascorbate level and higher succinic acid level, correlating with 5‐HT concentrations and increased crypt depth. Additionally, altered metabolic pathways (e.g., nucleotide metabolism, signal transduction, metabolism of cofactors and vitamins) suggested an impact on the colonic function. In summary, early inhibition of gut‐derived 5‐HT may unfavorably reshape the colonic microenvironment, affecting gut morphology, microbial function, stem cell proliferation, and mucosal metabolism.

## Highlights

- LP533401 significantly inhibited 5‐hydroxytryptamine (5‐HT) production in neonatal rats, altering colonic morphology by increasing crypt depth and decreasing myenteric thickness.
- Early‐life inhibition of peripheral 5‐HT altered colonic microbial functions, such as two‐component system and tyrosine metabolism, without affecting microbial composition.
- Overactivation of marker genes related to stem cell proliferation and the Wnt signaling pathway was correlated with crypt depth and 5‐HT level.

## Graphical abstract

The colonic microenvironment of neonatal rats was altered after administration of the peripheral 5‐hydroxytryptamine (5‐HT) inhibitor LP533401, resulting in increased crypt depth and decreased myenteric thickness. Multi‐omics approaches, including metagenomics, transcriptomics, and metabolomics, in combination with immunofluorescence, were employed to investigate the underlying mechanisms. With a decreased 5‐HT level, there was an increase in microbial two‐component system expression, pathogen infection‐responsive signaling, and Wnt signaling. Metabolite analysis showed increased succinic acid concentration and decreased pantothenic acid and ascorbate level. Additionally, the expression of Ascl2 and Wnt3, which promote stem cell proliferation, was significantly upregulated and positively correlated with increased crypt depth.

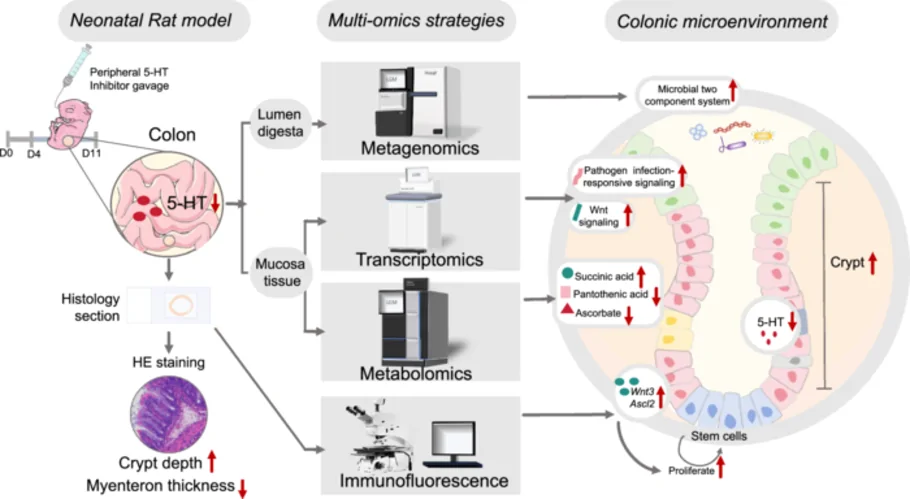

## 1 INTRODUCTION

5‐Hydroxytryptamine (5‐HT), known as serotonin, is a major neurotransmitter influencing both gut motility and brain function [1]. The majority of 5‐HT is produced in enterochromaffin cells (ECs) within the gastrointestinal tract, particularly in the proximal colon, while a minor percentage (~5%) is synthesized in the central nervous system [2, 3]. The synthesis of 5‐HT is facilitated by tryptophan hydroxylase (Tph) 1 and Tph2 [3]. Tph1‐dependent synthesis of 5‐HT is tightly linked to host tryptophan metabolism and regulated by the gut microbiome [4]. Previous studies have proven that gut microbes can regulate 5‐HT synthesis via acetate produced by *Lactobacillus amylovorus* [5]. Alterations in the 5‐HT signaling pathway have been found to affect intestinal function, including peristalsis and secretion [6, 7], primarily by activating 5‐HT receptors on nerve fibers and enteric neurons. For instance, knockout of 5‐HT receptor 4 in mice affects the development and survival of enteric neurons and promotes the generation of novel enteric neurons [8, 9]. The renewal of intestinal stem cells is also regulated by 5‐HT [10]. 5‐HT has been reported to promote the proliferation of intestinal stem cells and colorectal cancer cells [11, 12]. In embryos, 5‐HT is involved in controlling morphogenesis during developmental stages preceding the appearance of serotonergic neurons [13]. Neonatal mice deficient in *Tph1* showed significant functional impairment in alveolar and cardiovascular development, suggesting the indispensable role of 5‐HT in development [14].

The dysregulated intestinal microenvironment is a major cause of diseases and intestinal development disorders. The high turnover rate and constant renewal of the intestinal epithelium depend on intracellular signals, including Wnt and Notch ligands [15]. Moreover, the terminal differentiation of the enteric nervous system in the gut is influenced by the enteric microenvironment [16], which is also regulated by 5‐HT. Additionally, the gut microbiota could regulate the maturation of the enteric nervous system via enteric 5‐HT networks [17]. However, the comprehensive influence of 5‐HT on the early colonic microenvironment and its interplay with gut microbes remain unclear.

Peripheral 5‐HT synthesis, particularly in the gut, is associated with gastrointestinal symptoms, carcinoid tumors (carcinoid syndrome), obesity, and inflammation [18]. Understanding the impact of inhibiting peripheral 5‐HT synthesis may offer novel therapeutic avenues against these disorders. The neonatal phase is the key window of intestinal development and 5‐HT production in the enteric nervous system. This study aims to determine if an abnormality in 5‐HT synthesis affects gut homeostasis, which is fundamental to understanding the physiological relevance of gut‐derived 5‐HT. We hypothesize that dysregulation of gut‐derived 5‐HT affects the early colonic microenvironment. By employing LP533401 to inhibit gut‐derived 5‐HT synthesis in neonatal rats, this study evaluates the colonic microenvironment by assessing gut morphology, gut microbiome, and host function. Multi‐omics strategies have been widely used to understand host‐microbiome interaction in vivo [19, 20, 21], such as those in the colonic microenvironment of adult rats [20]. To study the gut microbiome and developmental profiles in neonatal rats, an integrative omics approach, including metagenomics, epithelial transcriptomics, and metabolomics was employed. The results indicate that inhibition of peripheral 5‐HT synthesis in early life disturbs the colonic microenvironment.

## 2 RESULTS

### 2.1 LP533401 gavage altered host tryptophan‐5‐HT metabolism

**Figure 1:**
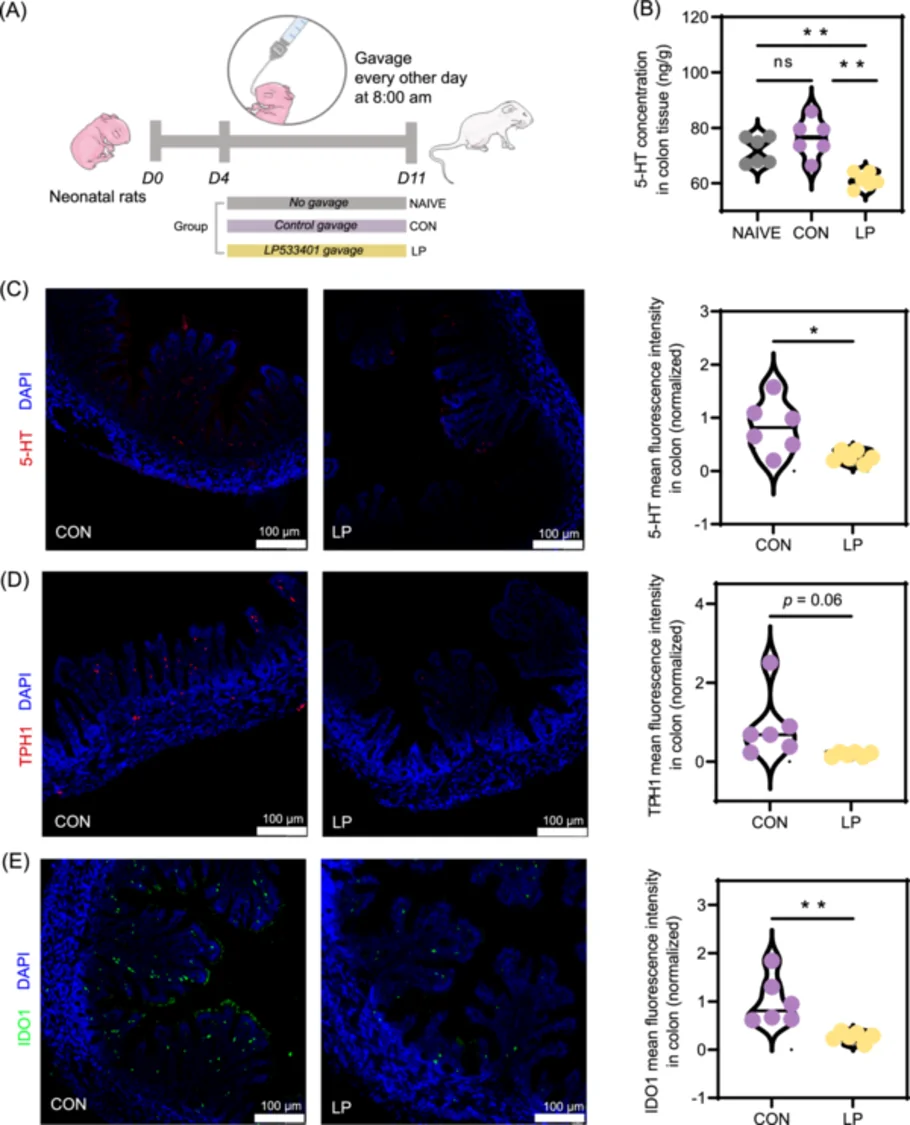
The influence of LP533401 on tryptophan‐5‐HT synthesis metabolism in the colon of neonatal rats. (A) Experimental design containing naïve (NAÏVE), control gavage (CON), and LP533401 gavage (LP) rat groups. (B) 5‐HT concentration in colon tissue. (C) Immunofluorescent imaging of 5‐HT in colonic sections. (D) Immunofluorescent imaging of TPH1 in colonic sections. (E) Immunofluorescent imaging of IDO1 in colonic sections. Left panel: Representative images (scale bar = 100 μm). Right panel: Quantification of mean fluorescence intensity. Data are shown as mean ± SEM, *n* = 6, ns means *p* > 0.05, *p < 0.05, **p < 0.01. 5‐HT, 5‐hydroxytryptamine; IDO1, indoleamine 2,3‐dioxygenase1; TPH1, tryptophan hydroxylase1.

To block the peripheral tryptophan‐5‐HT synthesis in early life, 18 neonatal rats were randomly assigned to three groups: naïve (NAÏVE, *n* = 6), control gavage (CON, *n* = 6), and LP533401 gavage (LP, *n* = 6) (Figure 1A). LP533401, an inhibitor of peripheral 5‐HT synthesis, was used to decrease peripheral 5‐HT levels [22, 23]. The rationale is to disturb Tyr235 and Phe241 (Figure S1) in the TPH1 catalytic site and thereby modulate Tph1 protein activity [23]. In the present study, there was no significant difference (*p* > 0.05) in colonic 5‐HT concentration between NAÏVE and CON groups (Figure 1B). However, LP533401 administration significantly reduced (*p* < 0.05) the colonic 5‐HT concentration compared to the NAÏVE and CON group (Figure 1B). Immunofluorescent analysis of colonic tissue further confirmed the reduced (*p* < 0.05) 5‐HT protein signal in the LP group (Figure 1C). Moreover, a downward trend (*p* = 0.06) in TPH1 fluorescent intensity (Figure 1D) and a significant decline (*p* < 0.05) in the fluorescent intensity of indoleamine 2,3‐dioxygenase (IDO1) (Figure 1E) were observed in the LP group. Taken together, these results validated the inhibition of colonic 5‐HT after LP533401 gavage.

### 2.2 Inhibition of peripheral 5‐HT affected colonic phenotype

**Figure 2:**
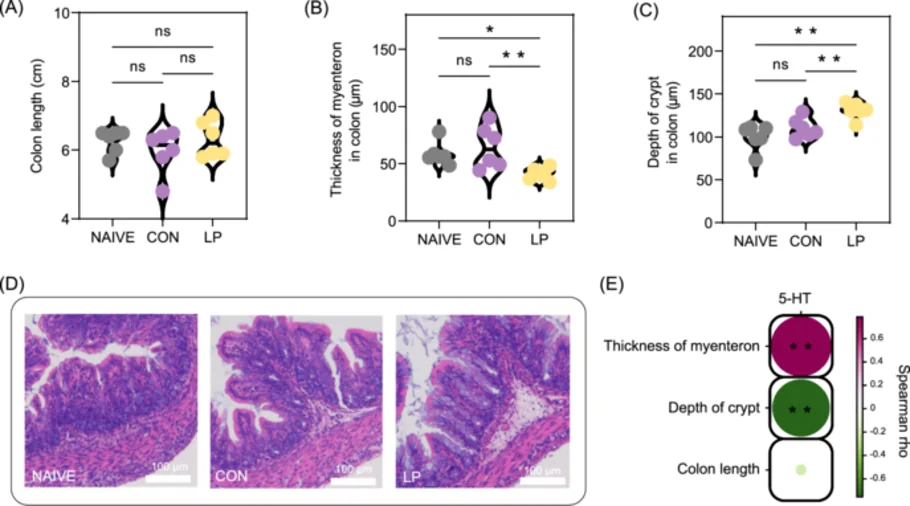
Colonic phenotype and the correlation with 5‐HT. (A) The length of the colon of rats in naïve (NAÏVE), control gavage (CON) and LP533401 gavage (LP) groups. (B) The quantitative analysis of myenteric thickness in the colon. (C) The quantitative analysis of colonic crypt depth. (D) Representative histological micrographs of the colon (scale bar = 100 μm). (E) Correlation analysis between colon phenotype and 5‐HT concentration. The asterisk indicates the significance of the Spearman correlation coefficient and describes the accuracy of the rho value. Data are shown as mean ± SEM, *n* = 6, ns means *p* > 0.05, *p < 0.05, **p < 0.01. 5‐HT, 5‐hydroxytryptamine.

Based on the premise of impaired intestinal 5‐HT synthesis, we investigated the colonic morphology in neonatal rats. There was no significant difference in colon length between groups (*p* > 0.05) (Figure 2A). Interestingly, the myenteron thickness was reduced (*p* < 0.05) and the crypt depth was increased in the colon of the LP group compared to NAÏVE and CON groups (Figure 2B,C). These results suggested the worsening morphology in the colonic environment after LP533401 gavage (Figure 2D). Furthermore, 5‐HT concentration was positively correlated with the thickness of the myenteron, and negatively correlated with crypt depth (*p* < 0.05, |rho| > 0.7; Figure 2E).

### 2.3 5‐HT inhibition slightly affected microbial function

**Figure 3:**
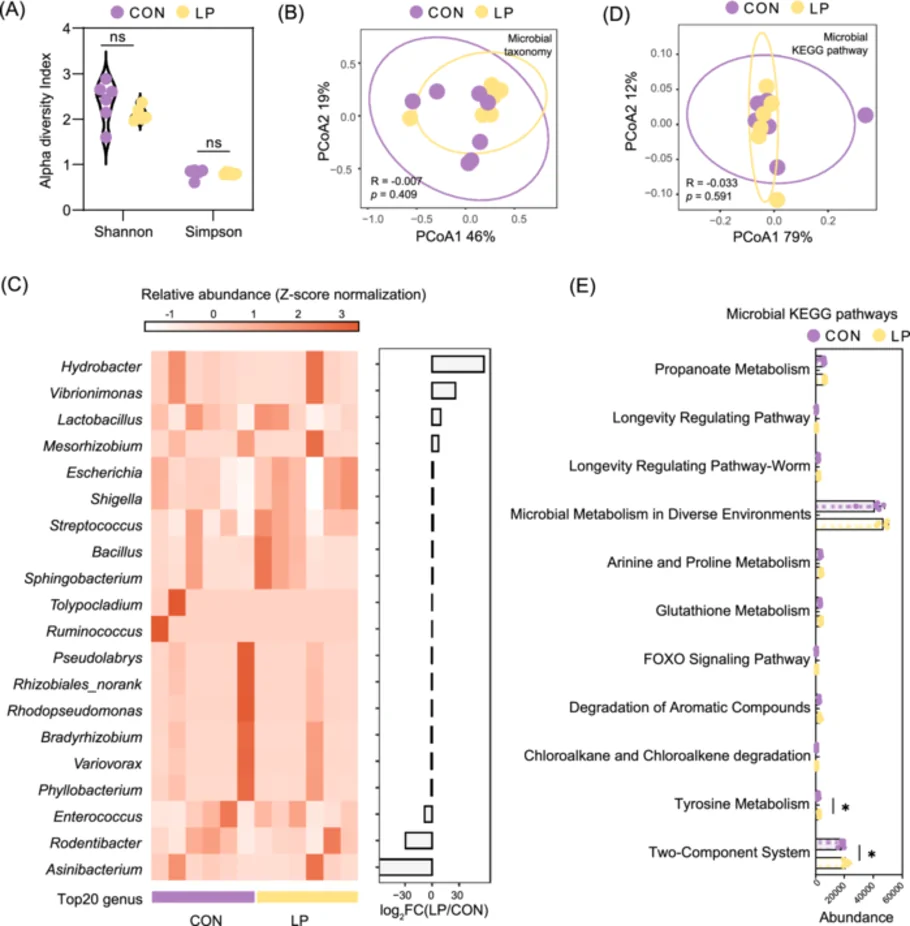
Metagenomics analysis of microbial composition and function in the colonic digesta. (A) Shannon and Simpson indices of colon microbiome between control gavage (CON) and LP533401 gavage (LP) rat groups. (B) Principal coordinate analysis (PCoA) analysis of the microbial taxonomy between CON group and LP group. (C) Relative abundance of top 20 microbial genera in the colon. (D) PCoA analysis of the microbial Kyoto Encyclopedia of Genes and Genomes (KEGG) pathways between CON and LP groups. (E) Relative abundance of enriched microbial KEGG pathways. Data are shown as mean ± SEM, *n* = 6, ns means *p* > 0.05, *p < 0.05.

To explore the impact on the microbiome, we examined the microbial composition and function in the colonic content via metagenome sequencing. Both the Shannon index and Simpson index were unchanged (*p* > 0.05), revealing that the microbial richness and evenness were stable after LP intervention (Figure 3A). The principal coordinate analysis (PCoA) analysis based on Bray‐Curtis distances showed that there was no difference of microbial community structure (*p* > 0.05) between CON and LP groups (Figure 3B). No differences were observed for the top 20 genera, including *Lactobacillus*, *Streptococcus*, and *Bacillus* (Figure 3C). To further investigate the impact on microbial function, we analyzed the enriched Kyoto Encyclopedia of Genes and Genomes (KEGG) pathways. Multivariable analysis based on the overall microbial functional readouts showed no differences between groups (*p* > 0.05) (Figure 3D). At the feature level, the relative abundance of the two‐component system (ko02020) and tyrosine metabolism (ko00350) pathways were higher (*p* < 0.05) in LP than the CON group (Figure 3E). Taken together, the colonic microbial composition was not affected but specific functions were altered by peripheral 5‐HT inhibition.

### 2.4 Transcriptomic analysis revealed altered epithelial functions

**Figure 4:**
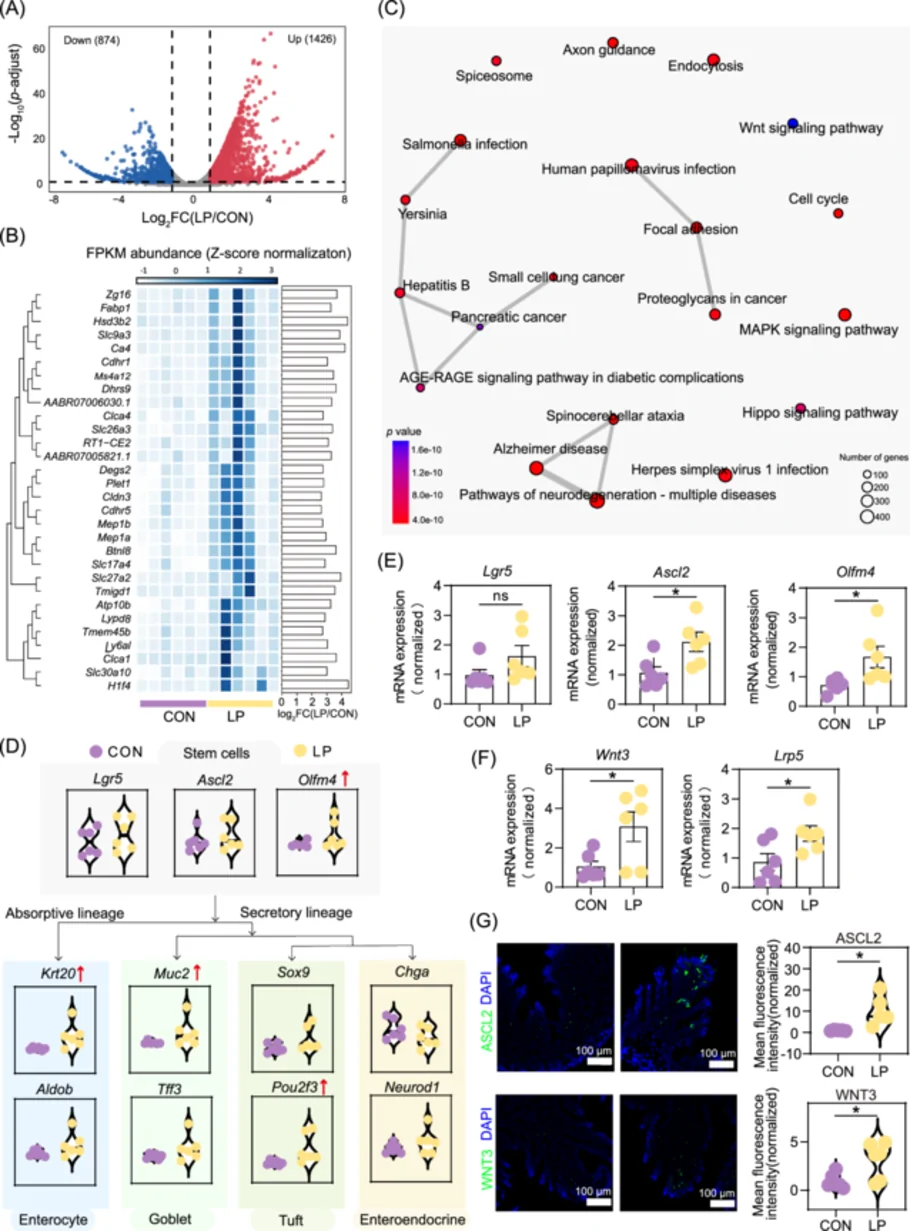
Transcriptomic analysis of colonic mucosa and mRNA expression of the marker genes related to the cell proliferation. (A) Volcano plot of differential genes between control gavage (CON) and LP533401 gavage (LP) rat groups. (B) Top 30 differential genes in heatmap. (C) Enriched transcriptomic pathways between CON group and LP group. (D) The abundance of marker genes related to cell proliferation and differentiation. (E) Relative mRNA expression of *Lgr5*, *Ascl2*, and *Olfm4* in colon tissue. (F) Relative mRNA expression of *Lrp5* and *Wnt3* in the colon tissue. (G) Immunofluorescence staining of ASCL2 and WNT3 proteins in the colon. Left panel: Representative images (scale bar = 100 μm). Right panel: Quantification of average fluorescence intensity. Data are shown as mean ± SEM, *n* = 6, ns means *p* > 0.05, *p < 0.05, ↑means upregulation, ↓means downregulation. *Aldob*, aldolase B; ASCL2/*Ascl2*, achaete‐scute complex homolog 2; *Chga*, chromogranin A; *Krt20*, keratin 20; *Lgr5*, leucine‐rich repeat‐containing G‐protein coupled receptor 5; *Lrp5*, low‐density lipoprotein receptor‐related protein 5; *Muc2*, mucin 2; *Neurod1*, neurogenic differentiation 1; *Olfm4*, olfactomedin 4; *Pou2f3*, POU class 2 homeobox 3; *Sox9*, SRY‐box transcription factor 9; *Tff3*, trefoil factor 3.

On top of the impact on the colonic microbiome, we further investigated the effect on the epithelial function via transcriptomics sequencing. As shown in the volcano plot (Figure 4A), a total of 874 genes were downregulated and 1426 genes were upregulated in the colonic tissue after LP533401 gavage at the criteria *p*‐adjust ≤ 0.05 and |log_2_ fold change (FC)| ≥ 2 (Figure 4A). LP group had higher expression of abundant genes, such as fatty acid binding protein 1 (*Fabp1*), solute carrier family 9 member A3 (*Slc9a3*), cadherin‐related family member 1 (*Chdr1*), and claudin 3 (*Cldn3*) (Figure 4B). Real‐time quantitative PCR (qPCR) was further used to validate the results of transcriptome sequencing, including the expression of tryptophan‐metabolizing enzymes and genes involved in stem cell signaling (Figure S2). Pathway enrichment of the discriminant genes analysis showed a neurology‐oriented triangle encompassing Alzheimer's disease, pathways of Neurodegeneration‐multiple disease, and Spinocerebellar ataxia (Figure 4C). Host metabolism pathways related to the pathogen infection‐responsive signaling (e.g., *Salmonella* and *Yersinia* infection) and cancer signaling (small cell lung cancer, proteoglycans in cancer, and pancreatic cancer) were altered after LP533401 gavage (Figure 4C). Furthermore, key pathways related to intestinal development, including the Wnt signaling pathway and the Hippo signaling pathway, were also enriched (Figure 4C). These results indicated that the inhibition of gut‐derived 5‐HT affected the epithelial function at the transcription level.

### 2.5 Marker genes related to cell proliferation and differentiation were changed

To study the possible mechanisms underlying the abnormal gut morphology related to cell proliferation and differentiation, the expressions of marker genes of different cell types in the transcriptome data set were analyzed (Figure 4D). The expression of olfactomedin 4 (*Olfm4*), a marker of stem cells [24], was upregulated in the LP group. The expression of keratin 20 (*Krt20*), a marker of absorptive enterocytes, was also elevated in the LP group. The secretory lineage includes goblet, tuft, and enteroendocrine cells. The maker gene for goblet cells, mucin 2 (*Muc2*), and for tuft cells, pou class 2 homeobox 3 (*Pou2f3*), were upregulated (Figure 4D). These results were further validated using qPCR analysis. The mRNA expression of *Olfm4* and achaete‐scute complex homolog 2 (*Ascl2*) mRNA expression was increased in the LP group (*p* < 0.05; Figure 4E). The expression of Wnt signaling pathway‐related genes, including low‐density lipoprotein receptor‐related protein 5 (*Lrp5*) and *Wnt3* [25], were also elevated in the LP group (*p* < 0.05; Figure 4F). The expression of ASCL2 and WNT3 was further verified at the cellular level using immunofluorescence staining of colon sections (*p* < 0.05; Figure 4G). Taken together, these results revealed that inhibition of peripheral 5‐HT synthesis might lead to the change of marker genes related to intestinal stem cell proliferation and Wnt pathway signaling.

### 2.6 Metabolomics analysis revealed key metabolites correlated with colonic phenotypes

**Figure 5:**
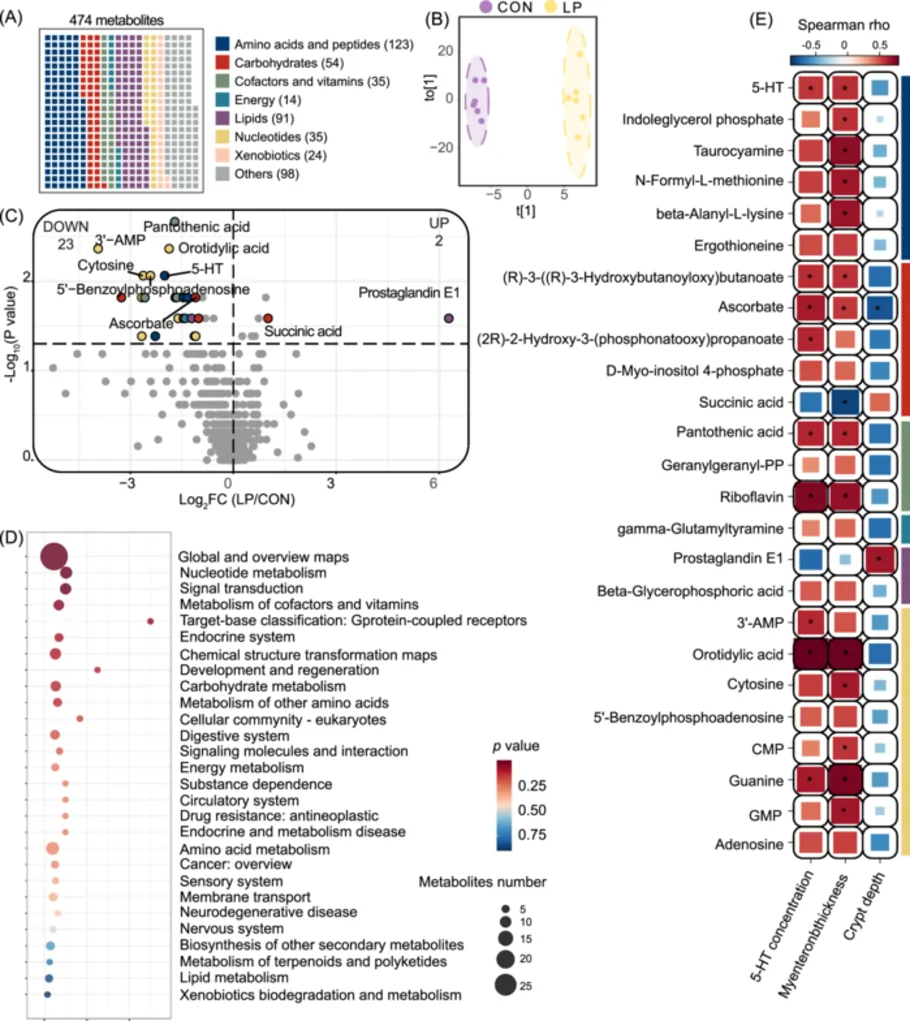
Metabolomics analysis of colon mucosa and the correlation with 5‐HT. (A) Classifications of 474 metabolites. (B) Orthogonal Partial Least Squares Discriminant Analysis (OPLS‐DA) of metabolome between control gavage (CON) and LP533401 gavage (LP) group. (C) Volcano plot of discriminating metabolites between groups. The color schemes of metabolite labels are consistent with the eight categories in panel A. (D) The enriched pathway of discriminant metabolites. (E) Correlation analysis between colonic phenotype and discriminating metabolites. The asterisk indicates the significance of the Spearman correlation coefficient and describes the accuracy of the rho value. The bar colors on the right side are consistent with the metabolite's eight categories.

A total of 474 metabolites were identified from the metabolome data set in the colonic mucosa (Figure 5A). These metabolites belong to eight major metabolic pathways, including amino acids and peptides, carbohydrates, cofactors and vitamins, energy, lipids, nucleotides, and xenobiotics. Orthogonal Partial Least Squares Discriminant Analysis (OPLS‐DA) showed a different metabolome structure between CON and LP groups (Figure 5B). A total of 25 distinguished metabolites were identified at *p* < 0.05 and variable importance projection (VIP) > 1.5. Among them, 23 were downregulated, including 5‐HT, pantothenic acid, cytosine, orotidylic acid, ascorbate, and 3′‐adenosine monophosphate (Figure 5C). Additionally, succinic acid and prostaglandin E1 were upregulated (Figure 5C). Metabolite pathway enrichment analysis showed the alteration in nucleotide metabolism, signal transduction, and the metabolism of cofactors and vitamins (Figure 5D). Furthermore, the correlations between discriminating metabolites and gut phenotype indices were established to reflect the underlying mechanism after 5‐HT inhibition. Notably, ascorbate, pantothenic acid, riboflavin, guanine, and orotidylic acid were positively correlated with 5‐HT concentration and the thickness of myenteron in the colon, and the succinic acid was negatively correlated with myenteron thickness (Figure 5E). Moreover, ascorbate was correlated with crypt depth negatively, while the prostaglandin E1 was associated with the depth of the colonic crypt positively (*p* < 0.05, |rho| > 0.7) (Figure 5E).

**Figure 6:**
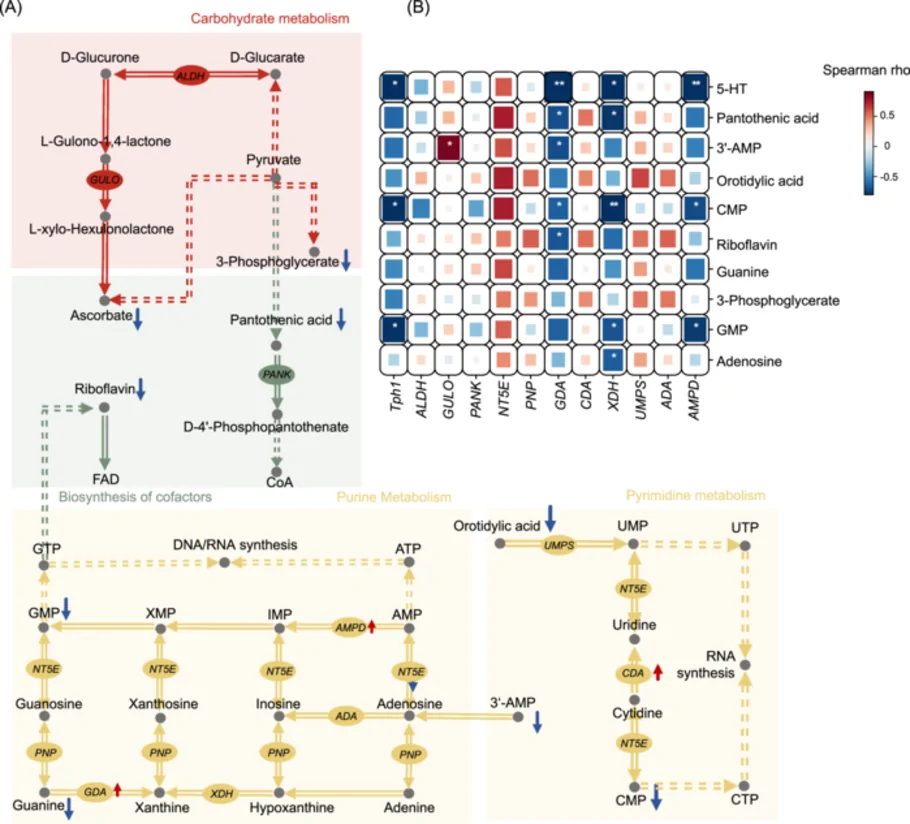
Integration of metabolome and transcriptome data set. (A) Maps of discriminating metabolites and related transcriptomic genes in metabolic process cluster. Arrows indicate changes in metabolite content: ↑ means upregulation, ↓means downregulation. (B) Correlation analysis between genes and metabolites. The asterisk indicates the significance of the Spearman correlation coefficient and describes the accuracy of the rho value. *ADA*, adenosine deaminase; *ALDH*, aldehyde dehydrogenase; *AMPD*, AMP deaminase; *CDA*, cytidine deaminase; *GDA*, guanine deaminase; *GULO*, l‐gluconolactone oxidase; *NT5E*, 5′‐nucleotidase; *PANK*, pantothenate kinase; *PNP*, purine nucleoside phosphorylase; *Tph1*, tryptophan hydroxylase 1; *UMPS*, uridine monophosphate synthetase; *XDH*, xanthine dehydrogenase

To elucidate the potential mechanism of how key metabolites were modulated at the transcription level, data from transcriptomics and metabolomics were integrated (Figure 6A). Among them, guanine deaminase (GDA) showed negative correlations with 5‐HT, pantothenic acid, and cytidine 3′‐monophosphate (CMP) (*p* < 0.05, |rho| > 0.7). Adenosine monophosphate deaminase (AMPD) correlated negatively with 5‐HT and guanosine monophosphate (GMP) (*p* < 0.05, |rho| > 0.7) (Figure 6B).

## 3 DISCUSSION

The present study aimed to elucidate the regulatory role of gut‐derived 5‐HT in the colonic microenvironment and gut microbiome in neonatal rats. These results demonstrated a significant impact of inhibiting gut‐derived 5‐HT synthesis on various aspects of intestinal morphology and metabolism.

Interaction between gut microbiota and 5‐HT has attracted wide attention due to their crucial roles in intestinal function. Our previous studies have proven that intestinal microbes can affect host 5‐HT production by regulating Tph1 enzyme activity [5]. However, the effect of host 5‐HT on the intestinal microbiota remains vague. It is worth noting that some microbes have also been reported to have receptors for sensing and transporting 5‐HT [26, 27]. Prior studies with *Tph1* knockedout mice indicated the potential effect of 5‐HT on intestinal microbiota [28]. However, due to the systemic knockout, the causative factors leading to the microbial alterations may be complicated beyond 5‐HT. In the present study, targeted inhibition of gut‐derived 5‐HT via LP533401 was employed to avoid other phenotypic changes caused by genetic knockout. The results showed that early inhibition of 5‐HT synthesis had minimal effects on day 11 microbiome in the colon. Contrary to the results in this study, another study showed that the genetic knockout of *Tph1* affects murine growth and microbial composition [28]. Differential findings may be due to the fact that the microbiome at 11 days of age in neonatal rats is still immature and less complex to observe a significant effect, which remains to be investigated in future studies.

Notably, even though the microbial composition has not been altered significantly, the microbial function was affected by LP533401 gavage. The microbial pathways of the two‐component system (ko02020) and tyrosine metabolism (ko00350) were upregulated. The expression of the two‐component system is reported to be upregulated in inflammatory bowel disease, indicating a negative association with intestinal health [29]. Moreover, this pathway serves as a basic mechanism for gut microbes to sense and respond to environmental changes, suggesting microbial reaction to the deficiency of 5‐HT. The hypothesis may be supported by an earlier study showing that specific microbes could sense alterations in surrounding 5‐HT [26].

Another interesting finding is the alteration of microbial tyrosine metabolism. Tyrosine and tryptophan metabolism are critical processes in regulating neurotransmitter synthesis. They share the common enzyme aromatic l‐amino acid decarboxylase, which converts l‐DOPA to dopamine and 5‐HTP (5‐hydroxytryptophan) to 5‐HT, respectively [30]. An earlier study proved that during (+)−3,4‐methylenedioxymethamphetamine‐induced 5‐HT depletion, local infusion of l‐tyrosine into the striatum or hippocampus worsens the dysfunction by reducing 5‐HT [31], suggesting the metabolic crosstalk between tyrosine and 5‐HT synthesis. Tyrosine and tryptophan could be utilized by gut microbes to produce secondary metabolites. However, whether alterations in microbial tyrosine metabolism affect host metabolism remains to be further explored.

The impact of 5‐HT on colonic stem cell proliferation and differentiation is another topic of interest in this study. Prior research has shown that 5‐HT produced from enteric serotonergic neurons can promote stem cell self‐renewal through Wnt/β‐catenin signaling [12]. Here, we observed that inhibition of peripheral 5‐HT production increased the marker gene expression of stem cells, revealing that there may be a potential relationship between 5‐HT, stem cells, and the Wnt signaling pathway. 5‐HT receptors are expressed widely in stem cells and other cell types to regulate proliferation and differentiation [4]. For example, 5‐HT_1B/1D/1F_ receptors in colorectal cancer stem cells can be engaged with Wnt/β‐catenin signaling [11]. Activation of 5‐HT_4_ receptors on goblet cells could promote Muc2 expression and improve colonic barrier function [32]. In the present study, LP533401 intervention led to increased expressions of marker genes of absorptive (e.g., enterocytes) and secretory (e.g., goblet cells and tuft cells) lineage cells, shedding light on altered colonic microenvironment due to 5‐HT deficiency.

Moreover, these changes in different intestinal cell types may be related to intestinal morphological changes. It has been reported that the knockout of 5‐HT_2A_ receptors resulted in a thinner muscle layer [33]. Similarly, the present study showed a lower thickness of myenteron after LP533401 intervention. It has been found that the Wnt signaling, which is located in the base of the crypt, serves to maintain stem cell proliferation and prevent differentiation [34]. In the present study, increased *Wnt3* and *Lrp5* expression revealed enhanced Wnt signaling in the colon. The increased *Ascl2* expression suggested promoted stem cell proliferation. However, abnormal Wnt signaling activation can lead to excessive cell proliferation and tumor formation [35]. Therefore, crypt depth may be increased in these lesions, reflecting abnormal cell proliferative activity in this study. Overall, inhibition of gut‐derived 5‐HT may affect the proliferation and differentiation of various types of cells, thereby affecting intestinal morphology.

5‐HT is a neuroendocrine peptide and plays a critical role in intestinal inflammation. Released 5‐HT can activate the 5‐HT_7_ receptor on dendritic cells to set off pro‐inflammatory cascades. Additionally, 5‐HT can also exert an anti‐inflammatory effect in the intestinal mucosa through 5‐HT_4_ receptors [36, 37]. In the present study, increased crypt depth and thinner intestinal wall indicate that the intestine has undergone significant inflammation and tissue damage. The IDO1‐mediated pathway was reported to exhibit a pro‐inflammatory effect in the body while IDO1 inhibition may reduce the damage caused by chronic inflammation [38]. However, our results showed that IDO1 mRNA and protein expression decreased significantly. It has been reported that there is a competitive relationship between *Tph1*‐mediated and *IDO1*‐mediated tryptophan metabolism [5], suggesting the 5‐HT intervention may inhibit IDO1 expression through negative feedback regulation.

Additionally, the present study showed the enrichment of pathogen infection pathways, including *Salmonella* infection, *Yersinia* infection, and Hepatitis B after 5‐HT inhibition, which is known to trigger significant intestinal inflammation through mechanisms involving the disruption of gut microbiota and activation of immune responses [39]. The downregulation of guanine in this study may support the hypothesis, as guanine has been reported to be involved in infectious diarrhea and colitis [40] and affect the formation of ligands for regulating Toll‐like receptor 9 [41]. At the metabolite level, increased succinic acid and decreased ascorbate may also provide evidence of intestinal inflammatory response. Succinic acid can enhance inflammation and play an important role in the host's energy homeostasis, regulating the composition of the intestinal microorganism [42, 43, 44]. Ascorbate is a key factor in inflammation, which can reduce oxidative stress and immune cell function. Crohn's patients are at risk for ascorbate deficiency, resulting in the pathogenesis of inflammatory bowel disease by altering the microbial composition [45]. Moreover, the pantothenic acid showed a decrease in the present study. Pantothenic acid could help alleviate inflammation by suppressing the JNK/P38 MAPK signaling pathway [46]. These findings indicate that the inhibition of peripheral 5‐HT synthesis is prone to pro‐inflammation response, but more evidence is needed to provide an in‐depth and comprehensive explanation in future studies.

To further explain the intrinsic causes and mechanisms by which inhibited 5‐HT altered gut development, a correlation analysis between 5‐HT, metabolites, and phenotypes was conducted. In this study, ascorbate, pantothenic acid, riboflavin, guanine, and orotidylic acid showed positive correlations with the thickness of colonic myenteron, on the premise that they were positively associated with 5‐HT concentrations. Consistent with the present findings, guanine is reported to be involved in the 5‐HT signal transduction process [47], which may be linked to the observed alteration in intestinal phenotype. It has been reported that the myenteron thickness is related to the digesta passage rate and nutrient digestibility [48]. The thinner bowel wall was found in chronic gut inflammation, especially in Crohn's disease or ulcerative colitis [49], and associated with deficiency of certain key nutrients such as vitamins [50]. Decreased ascorbate (vitamin C) may weaken the intestinal barrier, leading to increased intestinal wall permeability [51], which can trigger structural damage and thinning of the intestinal wall. Similarly, insufficient vitamins such as pantothenic acid may damage the intestinal integrity, while over‐supplementation of pantothenic acid reverses the process [52], providing a potential mechanism to explain the poor gut morphology observed in the present study.

The study is limited due to the animal replicates in neonates and the choice of testing only one time point at the end of the study. LP533401 may exert a dynamic regulation on the colonic function. This will be solved with future longitudinal studies. Whether early‐life intervention has long‐lasting effects remains unknown. Therefore, future investigation with more replicates and more time points will be helpful to explore how 5‐HT regulates the gut microenvironment in vivo.

## 4 CONCLUSION

In summary, inhibition of peripheral 5‐HT synthesis by LP533401 imposed significant effects on the colonic microenvironment in the neonatal rat model. The colonic histology was changed as reflected by the increased crypt depth and decreased myenteron thickness. Metagenomics analysis showed slightly altered microbial function but not microbial composition. Marker genes (e.g., *Ascl2* and *Wnt3*) in association with cell proliferation and the Wnt pathway were upregulated, mirroring the increased crypt depth after LP533401 intervention. The correlation between metabolites (e.g., ascorbate) with 5‐HT concentration and crypt depth may further explain the intricate interaction in the colon. Taken together, our results revealed that inhibition of gut‐derived 5‐HT may change the colonic microenvironment to an adverse tendency, which broadens our understanding of the regulatory role of 5‐HT in the colonic microenvironment.

## 5 METHODS

### 5.1 Experimental design

All animal procedures were approved by Nanjing Agricultural University and the University Laboratory Animal Welfare and Ethics Committee (Approval number: NJAU.No20220701141).

Sprague Dawley rats at 8 weeks old (body weight ~400 g) were obtained from Vital River. Animals were kept in solid plastic cages with constant conditions (temperature, 22°C; humidity, 62%) in a 12‐h‐light cycle. After 1‐week of acclimatization, males and females were paired following the breeding protocol. A total of 18 pups were obtained and randomly assigned to naïve (NAÏVE, *n* = 6), control gavage (CON, n = 6), and LP533401 gavage (LP, *n* = 6). All pups were nursed by their natural dams. Starting from 4 days of age, body weight was recorded every other day at 8:00 am, followed by oral gavage of LP533401 or an equal volume of solvent. LP‐533401 (MedChemExpress) was dissolved in PEG200: 5% dextrose solution at the ratio of 40:60 (v/v) to prepare a 2 mg/mL working solution. Based on previous studies, a dosage of 30 mg/kg was selected [53]. Naïve pups did not receive any treatment. Rats were euthanized by decapitation at postnatal day 11. The sample size was selected to minimize the use of newborn rats and balance the power of statistics.

Around 1 cm of the middle colon segment was cut off and stored in a tube with 4% paraformaldehyde at room temperature. The remaining colonic segment was cut vertically to obtain colonic digesta and colon tissue separately. Samples were flash‐frozen in liquid nitrogen and stored at −80°C until further analysis. The personnel performing the lab analysis were blinded to the grouping information of the study.

### 5.2 Measurement of 5‐HT levels

Colon tissue was washed with pre‐chilled phosphate‐buffered saline (PBS) (0.01 M, pH = 7.4) to remove residual blood, and the tissue was minced after weighing. The minced tissue was added with a volume ratio of 1:9 PBS and a small amount of 2 mm zirconium beads. To further lyse the tissue cells, the tissue samples were ground with a cell disrupter. The homogenate was centrifuged at 5000 × *g* for 5 min, and the supernatant was collected. The concentration of 5‐HT was detected with an ELISA kit (Mallbio).

### 5.3 Histology and immunofluorescence staining

Intestinal samples were fixed with 4% paraformaldehyde at room temperature. The tissue blocks were gradually dehydrated in a graded alcohol series and xylene to replace the alcohol in the tissue. The translucent tissue blocks were embedded in paraffin blocks, sliced into 5‐μm‐thick sections, and attached to glass slides. For histology, the sections were stained with hematoxylin–eosin (HE). All sections were observed and photographed under an optical microscope. The crypt depth and myenteric thickness of the colon were measured by Image‐Pro Plus 6.0.

For immunofluorescence staining, tissue sections were deparaffinized and rehydrated with xylene and descending ethanol gradients. Then the tissue antigen was repaired (99°C for 20 min in 10 mM sodium citrate, pH 6 solution) followed by 1 h of blocking at room temperature in the 5% bovine serum albumin (BSA). The sections were incubated with primary antibodies at 4°C overnight. To avoid nonspecific signals, sections were washed with PBS for 5 min, three times. The secondary antibodies were applied for 1 h at room temperature. The following antibodies were used: rabbit anti‐5‐HT antibody 1:200 (Bioss), rabbit anti‐TPH1 antibody 1:200 (Cell Signaling), mouse anti‐IDO1 antibody 1:200 (Proteintech), mouse anti‐ASCL2 antibody 1:200 (Proteintech), mouse anti‐WNT3 antibody 1:200 (Proteintech), FITC‐Goat Anti‐Mouse lgG (H + L) 1:200 (Elabscience). The 4′,6‐diamidino‐2‐phenylindole (DAPI) was used for the DNA fluorescence staining in nuclei. The images of all slices were taken on Leica Microsystems CMS GmbH (Leica Microsystems) for further analysis. The mean fluorescence intensity of 5‐HT, TPH1, IDO1, ASCL2, and WNT3 were analyzed by Image‐Pro Plus 6.0.

### 5.4 Metagenomics sequencing

Fecal genomic DNA in colonic digesta samples was extracted with the E.Z.N.A.® stool DNA Kit (Omega Bio‐Tek). One percentage of agarose gel electrophoresis was used to detect the quality of extracted fecal genomic DNA. Covaris S220 Focused‐ultrasonicator (Woburn) was employed to shear the 1 μg genomic DNA from each sample. The DNA library was then constructed according to the manufacturer's instructions (Biozeron). All samples were sequenced in the pair‐end 150 bp mode on the Illumina HiSeq X instrument. To remove contaminants of adapter and low‐quality reads, Trimmomatic (parameters: ILLUMINACLIP: adapters. fa:2:30:10 SLIDINGWINDOW:4:15 MINLEN:75, http://www.usadellab.org/cms/uploads/supplementary/Trimmomatic) was employed on these raw sequence reads. Quality‐controlled reads were mapped to the rat genome (mRatBN7.2/rn7) by the BWA mem algorithm (parameters: ‐M ‐k 32 ‐t 16, http://bio-bwa.sourceforge.net/bwa.shtml). In the final step, clean reads have been filtered and trimmed to avoid host‐genome contamination and low‐quality data and then used for further analysis. A taxonomy of clean reads was determined based on the customized Kraken database, containing all bacteria, archaea, fungi, viruses, protozoa, and algae genome sequences in the NCBI RefSeq database. The abundance of taxonomy was estimated by Bracken (https://ccb.jhu.edu/software/bracken/). A set of contigs from a clean sequence reads with “‐‐min‐contig‐len 500” parameters with the help of MegaHit. The open reading frames (ORFs) of assembled contigs were predicted by using Prodigal (v2.6.3). Then, a set of unique genes from ORFs after clustering using CD‐HIT was obtained (parameters: ‐n 9 ‐c 0.95 ‐G 0 ‐M 0 ‐d 0 ‐aS 0.9 ‐r 1). The longest sequence of each cluster was considered as the representative sequence of each gene in the unique gene set and the gene abundance was calculated using salmon software. To identify the proteins to retrieve their function and annotations, BLASTX was used, and the results were annotated according to the unique gene set in KEGG databases. According to the KO results of colon digesta samples, the KEGG Pathway database was used to determine the specific functions and pathways of each sample by annotating the pathways mapped by genes.

### 5.5 RNA extraction and real‐time quantitative PCR

The total RNA was extracted from the colonic tissue as described below. The tissue samples were drowned in cold Trizol and then ground into a suspension with zirconium bead milling. The suspension was centrifuged at 12,000 × *g* for 5 min at 4°C and removed the supernatant. 1/5 volume of chloroform was added to the supernatant and shaken vigorously for 15 seconds to make an emulsion. The emulsion was centrifuged at 12,000 × *g* for 15 min at 4°C, and the upper aqueous phase liquid was removed to another tube. Then, an equal volume of pre‐cooled isopropanol was added and mixed with liquid. The liquid was centrifuged at 12,000 × *g* for 10 min at 4°C. After centrifugation, the upper liquid was discarded and the bottom precipitate was retained. 1 ml of 75% ethanol (prepared with RNase‐free double‐distilled water) was added to suspend the precipitate. Then the ethanol was centrifuged at 12,000 × *g* for 5 min at 4°C. The precipitate RNA was dried for 5 min and then dissolved in RNase‐free water to test for purity, concentration, and integrity.

All cDNA was synthesized from RNA using the HiScript III RT SuperMix (Vazyme). RT‐PCR was performed using the ChamQ Universal SYBR Green qPCR Master Mix (Vazyme) run on Applied Biosystems QuantStudio 5 PCR System (Thermo Fisher Scientific). Fold changes were calculated using the ΔΔCT method. The mRNA expression was normalized to the expression of *β‐actin* as the internal reference gene, and the method of calculation was based on previous studies [54]. All primer sequences are listed in Table S1.

### 5.6 Nontargeted metabolomics

The samples were sent to Panomix for metabolome detection. The colonic tissue sample and the steel beads were submerged in the tissue extraction solution, and centrifuged at 12,000 × *g* for 10 min at 4°C. The supernatant was extracted for performing LC‐MS detection. LC analysis was performed with Thermo Vanquish (Thermo Fisher Scientific) Ultra Performance Liquid Systems with ACQUITY UPLC ® HSS T3 T3 (2.1 × 150 mm, 1.8 µm) (Waters) column. Mass spectrometric detection of metabolites was performed on Orbitrap Exploris 120 (Thermo Fisher Scientific) with an ESI ion source. Simultaneous MS1 and MS/MS (Full MS‐ddMS2 mode, data‐dependent MS/MS) acquisition were used.

The raw data were firstly converted to mzXML format by MSConvert in the ProteoWizard software package (v3.0.8789) [55] and processed using XCMS [56] for feature detection, retention time correction, and alignment. The metabolites were identified by accuracy mass (<30 ppm) and MS/MS data which were matched with HMDB [57] (http://www.hmdb.ca), massbank [58] (http://www.massbank.jp/), LipidMaps [59] (http://www.lipidmaps.org), mzcloud [60] (https://www.mzcloud.org) and (KEGG [61]. The robust LOESS signal correction (QC‐RLSC) [62] was applied for data normalization to correct for any systematic bias. After normalization, only ion peaks with relative standard deviations (RSDs) less than 30% in QC were kept to ensure proper metabolite identification.

Differential metabolites were subjected to pathway analysis by MetaboAnalyst [63], which combines results from powerful pathway enrichment analysis with the pathway topology analysis. The identified metabolites in metabolomics were then mapped to the KEGG pathway for biological interpretation of higher‐level systemic functions. The metabolites and corresponding pathways were visualized using the KEGG Mapper tool. The metabolic pathways were analyzed using MetaboAnalyst 4.0 (https://www.metaboanalyst.ca/).

### 5.7 Transcriptomic sequencing

The total RNA of colon tissue was extracted with the TRIzol® Reagent (Invitrogen) according to the details in the “RNA extraction and real‐time quantitative PCR” section. The genomic DNA was removed using DNase I (Takara). RNA quality was then determined using the 2100 Bioanalyser (Agilent) and quantified using the ND‐2000 (NanoDrop Technologies). Sequencing library was constructed with High‐quality RNA sample (OD_260/280_ = 1.8–2.2, OD_260/230_ ≥ 2.0, RIN ≥ 6.5, 28S:18S ≥ 1.0, > 10 μg).

One microgram of total RNA was employed to prepare RNA‐seq transcriptome libraries, using a TruSeq™ RNA sample preparation Kit from Illumina. The messenger RNA was isolated with polyA selection by oligo (dT) beads, then fragmented employing fragmentation buffer. All the cDNA synthesis, end repair, A‐base addition and ligation of the Illumina‐indexed adapters were performed according to Illumina's protocol.

The libraries were then size‐selected on 2% Low Range Ultra Agarose for cDNA target fragments of 200–300 bp, and PCR amplified using Phusion DNA polymerase (NEB) for 15 PCR cycles. The paired‐end libraries were sequenced by Illumina NovaSeq. 6000 sequencing (150 bp*2, Shanghai Biozeron Co., Ltd.). The trimmomatic with parameters (SLIDING WINDOW: 4: 15 MINLEN: 75) (version 0.36 http://www.usadellab.org/cms/uploads/supplementary/Trimmomatic) was employed to trim and quality control the raw paired‐end reads, then the clean reads were separately aligned to reference genome with orientation mode using hisat2 (https://ccb.jhu.edu/software/hisat2/index.shtml) software. This software was used to map with default parameters. The qualimap_v2.2.1 (http://qualimap.bioinfo.cipf.es/) was used to process the quality assessment of data. The gene reads were counted with htseq (https://htseq.readthedocs.io/en/release_0.11.1/).

### 5.8 Statistics and visualization

Power calculations indicated a sample size of 5 rats per treatment group could detect an effect size D of 2.54 with 95% power and a type I error of 5% for a one‐tail *t*‐test using the G*Power 3.1.9.7. All data were analyzed by R 4.3.1 [64, 65], and visualized by using R 4.3.1 [65, 66], GraphPad Prism 9 [67], and OmicStudio tools [68].

For 5‐HT concentration and HE staining analysis, the one‐way ANOVA was used to detect significant differences for multiple comparisons with Fisher's LSD test. For Immunofluorescence and qPCR analysis, the Student's t‐test was used to detect significant differences between two groups. The *p*‐value < 0.05 was considered statistically significant. For the microbiota data, the Wilcoxon signed‐rank test was used to detect significant differences between the two groups. *p* < 0.05 was considered statistically significant. For the transcriptomics data, the edgeR was used to screen the statistically differential genes between groups under the criteria (*p*‐adjust ≤ 0.05 and |log_2_ FC| ≥ 2). For metabolomics data, OPLS‐DA was used to determine the statistically discriminating metabolites under the criteria (*p* < 0.05 and VIP value > 1.5). The correlation analysis was calculated by the Spearman method. The values with *p* < 0.05 and |rho| > 0.7 were considered to be significant correlations.

The amino acid sequence of the Tph1 protein was acquired from NCBI (NP_004170.1). The protein structure model was built with the SWISS‐MODEL website [69]. Specific amino acid residues were marked in the DeepView software [70].

## AUTHOR CONTRIBUTIONS

**Weiyun Zhu and Chunlong Mu:** Conceived, designed, and supervised this study. **Yidan Ling and Ziyu Liu:** Conducted the experiment, wrote and modified the manuscript and performed statistical tests. **Shuibing Han and Haiqin Wu:** Helped the animal experiments and revised the manuscript. All authors have read and approved the final manuscript.

## CONFLICT OF INTEREST STATEMENT

The authors declare no conflict of interest.

## ETHICS STATEMENT

All animal procedures were approved by the Nanjing Agricultural University and Institutional Laboratory Animal Welfare and Ethics Committee (approval number: NJAU. No. 20220701141).

## Supporting information


**Figure S1:** Amino acid site of Tph1 protein structure and the sites of action by LP. Figure S2 qPCR verification results of differential genes in transcriptome.

**Table S1:** Primer Sequence for qPCR.

## Data Availability

The assembly data from metagenomics sequencing have been deposited at the NCBI Sequence Read Archive (SRA) database (BioProject: PRJNA1062915, at https://www.ncbi.nlm.nih.gov/bioproject/PRJNA1062915). The assembly data from transcriptome sequencing have been deposited at the NCBI Sequence Read Archive (SRA) database (BioProject: PRJNA1063604, at https://www.ncbi.nlm.nih.gov/bioproject/ PRJNA1063604/). Supplementary materials (figures, tables, graphical abstracts, slides, videos, Chinese translated versions, and updated materials) may be found in the online DOI or iMeta Science http://www.imeta.science/imetaomics/. The data and scripts used are saved in GitHub https://github.com/LingYD/2409Serotonin/tree/main.
