# Supplementary material for: Integrated omics revealed the altered colonic microenvironment after inhibition of peripheral serotonin synthesis by LP533401
Source: IMetaOmics. 2024 Oct 14;1(2):e34. doi: 10.1002/imo2.34 (PMC12806301; doi:10.1002/imo2.34)
Supplement: Supplementary file 1 — Figure S1: Amino acid site of Tph1 protein structure and the sites of action by LP. Figure S2 qPCR verification results of differential genes in transcriptome. [file IMO2-1-e34-s001.docx]

**Supporting information to**

**Integrated omics revealed the altered colonic microenvironment after inhibition of peripheral serotonin synthesis by LP533401**

**Running title:** Peripheral serotonin in colonic homeostasis

Yidan Ling^1,2 #^, Ziyu Liu^1,4 #^, Shuibing Han^1,2^, Haiqin Wu^1, 2^, Chunlong Mu^3^*, Weiyun Zhu^1,2^*

^1^Laboratory of Gastrointestinal Microbiology, College of Animal Science and Technology, Nanjing Agricultural University, Nanjing 210095, China

^2^National Center for International Research on Animal Gut Nutrition, National Experimental Teaching Demonstration Center of Animal Science, Nanjing Agricultural University, Nanjing 210095, China

^3^Department of Biochemistry and Molecular Biology, Cumming School of Medicine, University of Calgary, Calgary AB T2N 1N4, Canada.

^4^Department of Animal Science, University of Arkansas, Fayetteville 72701, USA

^#^These authors contributed equally: Yidan Ling, Ziyu Liu

*Correspondence: [chunlong.mu1@ucalgary.ca](mailto:chunlong.mu1@ucalgary.ca) (Chunlong Mu) and zhuweiyun@njau.edu.cn (Weiyun Zhu)

**Supplementary figure**


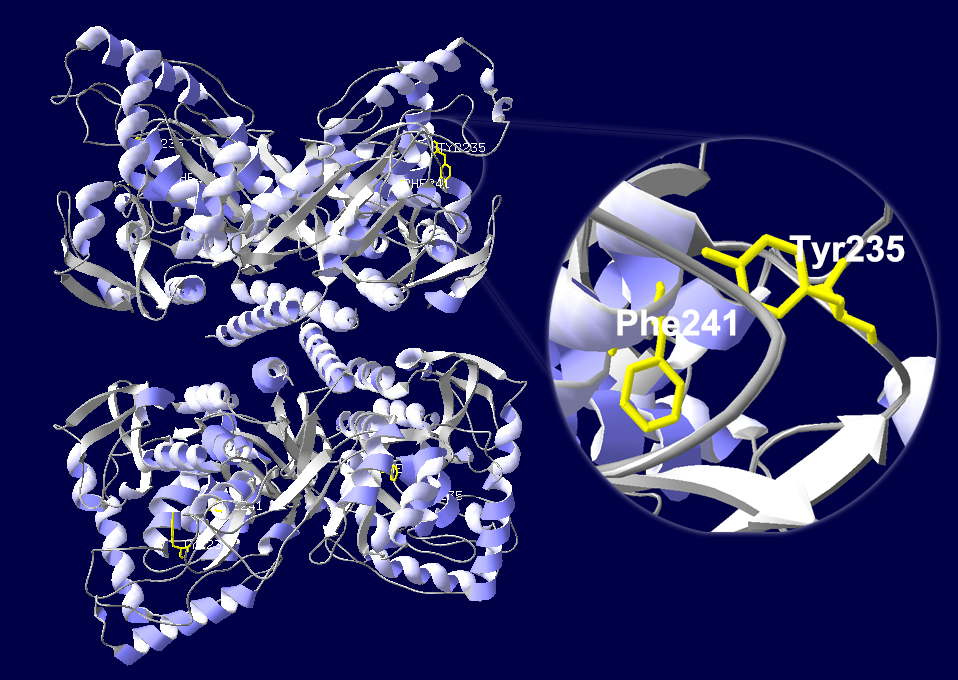


**Figure S1 Amino acid site of TPH1 protein structure that LP acts on Mechanism of LP533401 work: engages with Tyr235 and Phe241 to prevent TPH1 activity.**


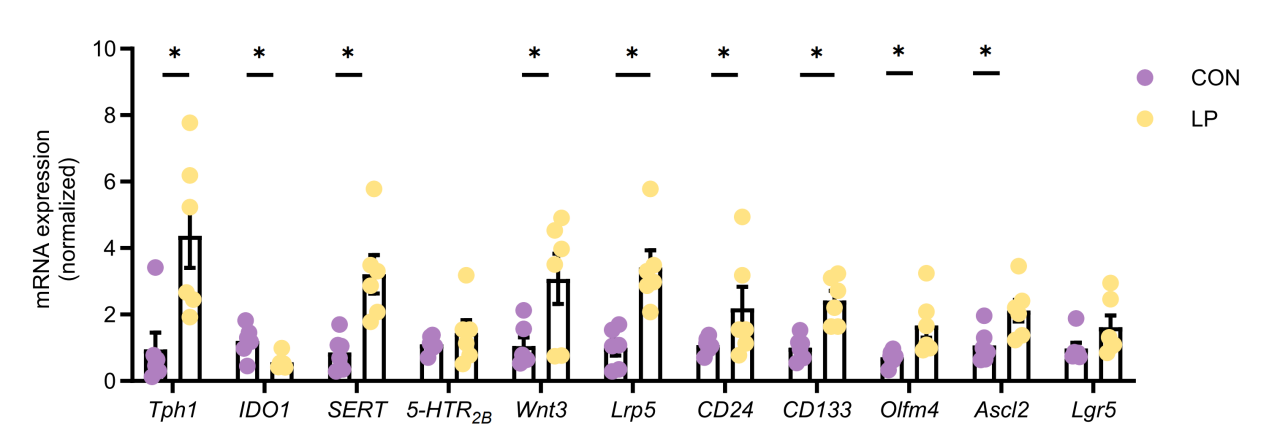


**Figure S2 RT-PCR verification results of differential genes in transcriptome.** Relative mRNA expression of some differential genes in transcriptome. Data are shown as mean ± SEM, n = 6, ns means *p* > 0.05, **p* < 0.05.
